# Teaching brain-machine interfaces as an alternative paradigm to neuroprosthetics control

**DOI:** 10.1038/srep13893

**Published:** 2015-09-10

**Authors:** Iñaki Iturrate, Ricardo Chavarriaga, Luis Montesano, Javier Minguez, José del R. Millán

**Affiliations:** 1 Instituto de Investigación en Ingeniería de Aragón, Dpto. de Informática e Ingeniería de Sistemas, Universidad de Zaragoza, Spain; 2 Defitech Chair in Brain-Machine Interface, Center for Neuroprosthetics & Institute of Bioengineering, School of Engineering, Ecole Polytechnique Fédérale de Lausanne, CH-1015 Lausanne, Switzerland

## Abstract

Brain-machine interfaces (BMI) usually decode movement parameters from cortical activity to control neuroprostheses. This requires subjects to learn to modulate their brain activity to convey all necessary information, thus imposing natural limits on the complexity of tasks that can be performed. Here we demonstrate an alternative and complementary BMI paradigm that overcomes that limitation by decoding cognitive brain signals associated with monitoring processes relevant for achieving goals. In our approach the neuroprosthesis executes actions that the subject evaluates as erroneous or correct, and exploits the brain correlates of this assessment to learn suitable motor behaviours. Results show that, after a short user’s training period, this teaching BMI paradigm operated three different neuroprostheses and generalized across several targets. Our results further support that these error-related signals reflect a task-independent monitoring mechanism in the brain, making this teaching paradigm scalable. We anticipate this BMI approach to become a key component of any neuroprosthesis that mimics natural motor control as it enables continuous adaptation in the absence of explicit information about goals. Furthermore, our paradigm can seamlessly incorporate other cognitive signals and conventional neuroprosthetic approaches, invasive or non-invasive, to enlarge the range and complexity of tasks that can be accomplished.

Research on brain-machine interfaces (BMI) has demonstrated how subjects can voluntary modulate brain signals to operate neuroprosthetic devices^1^,^2^,^3^,^4^,^5^,^6^. These BMIs typically decode cortical correlates of movement parameters (velocity/position^1^,^5^,^6^,^7^,^8^,^9^ or muscular activity^4^) in order to generate the sequence of movements for the neuroprosthesis. This control approach directly links neural activity to motor behaviour^10^. Mounting evidence, however, seems to suggest that motor control is the result of the combined activity of the cerebral cortex, subcortical areas and spinal cord. In fact, many elements of skilled movements, from manipulation to walking, are mainly handled at the brainstem and spinal cord level with cortical areas providing an abstraction of the desired movement such as goals and movement onset^11^. A BMI can mimic this principle, as studies have shown the feasibility to decode such a kind of cognitive information associated with voluntary goal-directed movements^3^,^12^,^13^. As an advantage of this approach over typical BMIs, and once the individual decoders are learnt, subjects do not need to learn to modulate their brain activity in order to generate all necessary movement parameters to operate the neuroprosthesis, which imposes natural limits on the complexity of tasks that can be solved. Nevertheless, this approach requires an intelligent neuroprosthesis, emulating the roles of the subcortical areas and spinal cord, capable to learn and generate the desired behaviours.

Here we demonstrate this alternative teaching paradigm (Fig. 1), where the neuroprosthesis learns optimal motor behaviours (or control policies) to reach a target location based on the decoding of human brain signals that carry cognitive information about the appropriateness of goal-directed movements —i.e., the error-related potential (ErrP)^14^,^15^, a time-locked potential elicited when actions do not match users’ expectations^16^,^17^,^18^,^19^,^20^,^21^. Error-related signals have been recently used to correct or adapt BMI decoders using both invasive^22^,^23^ and non-invasive recordings^15^,^24^. In our paradigm error information is exploited to learn a motor behaviour that accomplishes the user’s intended task from a set of basic pre-programmed actions. The user monitors the performance of the neuroprothesis as it executes a sequence of these actions. ErrPs are evoked by actions that the user considers wrong to achieve his/her desired goals, decoded online, and employed as a reward signal for a reinforcement learning algorithm (RL)^25^ that improves the neuroprosthesis behaviour. We tested this approach in three closed-loop experiments of increasing real-life applicability involving twelve subjects (Fig. 2a). They ranged from 1D cursor movement, to a simulated robot, and, finally, a real robot arm—both robots operating in a 2D space.

Each experiment consists of two phases: training the ErrP decoder from the user’s electroencephalogram (EEG) signals and online operation of the neuroprosthesis which, using the trained decoder, learns different reaching tasks. To train the decoder, each subject observed around 350 robot movements (or device actions) while it tries to reach predefined targets with 20% of wrong actions (i.e., movements away from the target location). During online operation, subjects, but not the neuroprosthesis, knew the target location and monitored the performance of the device. One run, lasting 100 device actions, was performed for each possible target (i.e., circles in Fig. 2a). The device controller was initialized to a random behaviour (i.e., equiprobable actions for all states) at the beginning of each run, and updated after each action based on the online decoding of the ErrPs. Whenever the device reached the target, the former was randomly reset to a new location. For Experiments 2 and 3, there were two targets (practice targets) that were used during ErrP calibration and online operation; and two targets (new targets) that were only used during online operation.

## Results

### Decoding error-related EEG potentials

**Figure 2:**
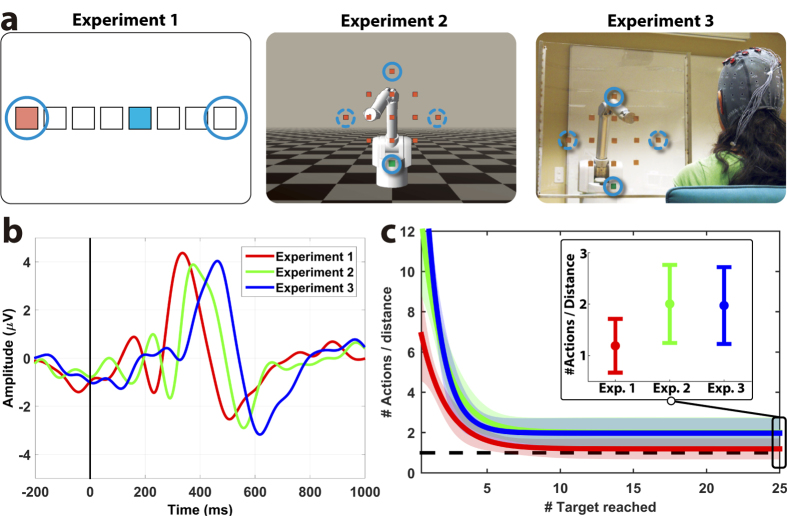
Learning optimal behaviours from error-related brain activity. (**a**) Experimental setup. In Experiment 1, the device (blue square) can move one position to the left or to the right in order to reach the target (red square). In Experiments 2 and 3, the robot moves left, right, up, or down across 13 states (orange squares) to reach a target (green square). Solid and dashed circles denote practice and new targets, respectively. (**b**) Grand-average difference event-related potentials (ERP) for each experiment during the training phase at channel FCz (N = 12); t = 0 ms represents the action onset. This difference ERP is computed as the difference of the subjects’ evoked EEG response after erroneous and correct actions of the device. (**c**) Normalized number of actions needed to reach the targets within a run. Lines correspond to the fitting of an exponential function to the data of each experiment, with the 95% confidence interval shown as shadows of the fitting line (all subjects combined). The horizontal line (Y = 1) indicates the optimal performance (See Methods). The inset shows the mean (±the 95% confidence) convergence value of the curve for each experiment.

**Figure 3:**
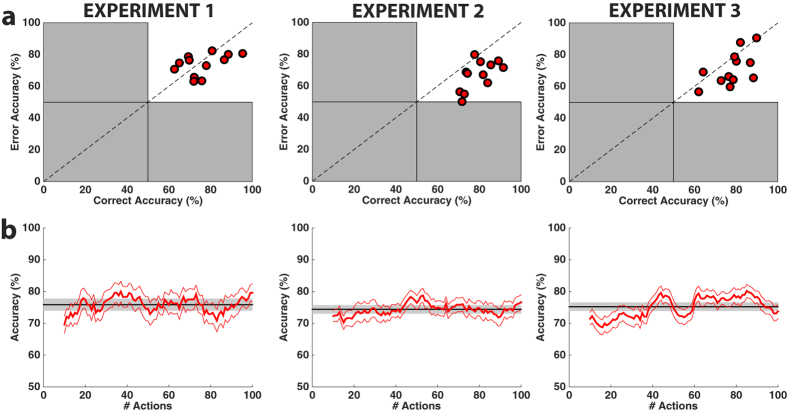
Online classification accuracy. (**a**) For each subject and experiment, ErrP online classification accuracy. The x-axis and y-axis represent the correct and error accuracies, respectively. Each dot corresponds to the average online accuracy achieved by each subject. (**b**) Decoding performance throughout the RL execution. For each experiment, decoding performance (mean ± SEM, thick ± thin red lines) throughout one run, where x-axis represents the actions along the run. The performance is computed as the accuracy obtained in a sliding window of 10 actions. The black horizontal line indicates the accuracy for each experiment, with the SEM shadowed. Results are averaged across runs (2, 4 and 4 for experiments 1, 2 and 3 respectively), and across subjects (N = 12). Confidence intervals (α = 0.05) for the accuracies throughout the run were of [71.30, 80.47], [70.93, 77.84], [68.67, 81.52] for experiments 1 to 3, respectively. The results showed no substantial differences in the accuracy variability throughout the runs.

ErrPs elicited in all protocols were consistent with previous studies^20^,^21^. The difference event-related potential (ERP) for erroneous and correct actions of the device exhibited a characteristic waveform with prominent fronto-central positive and negative peaks at around 300 and 500 ms, respectively. Figure 2b shows these ERPs at electrode FCz for all subjects in the three experiments. Statistically significant effects were observed on the latency but not the amplitude of these ERPs^26^,^27^ (see Supplementary Material). Accuracy of online single-trial decoding of the ErrPs was comparable for all experiments, independently of the of the task performed. Classification performance (73.8%, 72.5%, 74.3% on average for Experiments 1 to 3 respectively) exceeded the chance level (except for one subject in Experiment 2, see Fig. 3a)—a necessary condition for a reinforcement learning system to acquire an optimal control policy^25^. Remarkably, this decoding performance remained similar to the overall accuracy (FDR-corrected two-tailed independent t-test, p > 0.05) during the whole experiment (see Fig. 3b) despite the fact that the neuroprotheses move randomly at the beginning of an experiment and the error rate decreases as the devices learn an optimal motor behaviour (i.e., control policy).

To discard the influence of artifacts on the ErrP decoding, the data used to train the classifier included all possible movements for each class, thus reducing the possibility that classification was biased by their directions. For instance, during Experiment 1, both targets are used for the classifier training, thus the error and correct assessments are not likely to be correlated with left or right eye movements. Moreover, results obtained when testing new targets in Experiments 2 and 3 further support the fact that ErrP classification depends on the movement evaluation and not on its direction. Indeed, the training set only contained samples where the target locations were Up and Down, while the BMI was also tested on targets Left and Right. Finally, to assess whether the trained classifier discriminated different directions rather than assessments, we computed for each subject the accuracy of decoding the different pairs of movement directions (e.g., left versus right, up versus left, …) from a fixed assessment (either correct or erroneous) with the same features and classifier used during the experiments. The mean accuracies obtained were of 52.16 ± 5.22, 50.07 ± 5.07, and 49.48 ± 6.41 for Experiments 1 to 3, and thus did not exceed the chance levels of 56%, 54% and 54% (see Methods, ‘ErrP classifier’), proving that the classifier was not trained to distinguish movement directions or associated ocular artifacts, but user’s assessments. Additionally, statistical analyses of grand average ERPs shows significant differences only for assessment, but not for movement direction (see Methods and Supplementary Fig. S1).

In summary, our ERP analysis supports the hypothesis that ErrPs reflect a common phenomenon across all experiments, where the protocol mainly affects the temporal characteristics of the brain response.

### ErrP-mediated acquisition of control policies

**Figure 4:**
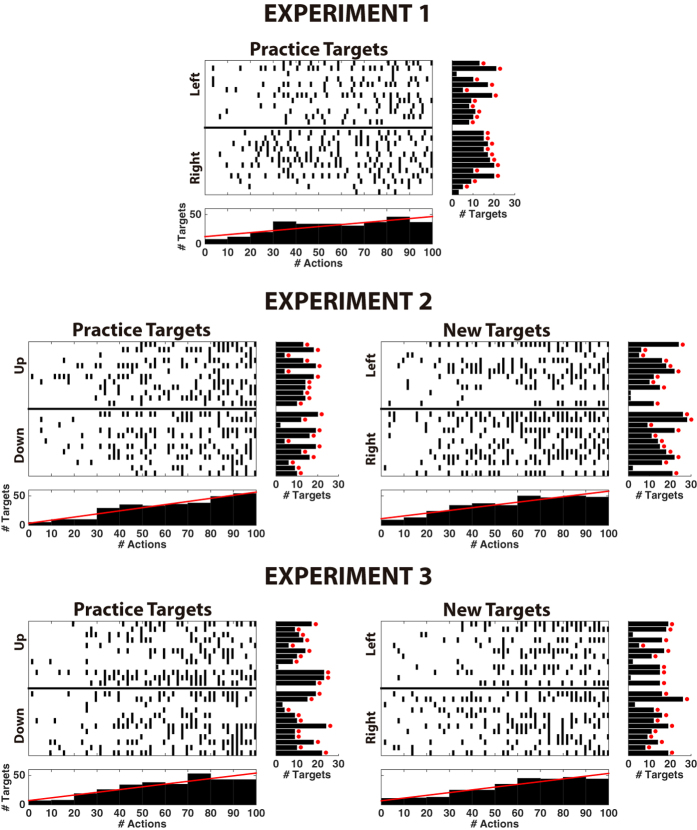
Number of reached targets within a run. Each subfigure corresponds to either practice or new targets for each experiment. For each subfigure: (Top) Raster plot, where x-axis represents time within a run (from 1 to 100 actions performed by the device), and each row in the y-axis represents one of the 12 subjects for each of the two targets. Every tick corresponds to the moment a target is reached. (Bottom) Histogram (bin size of 10 actions) associated with the upper raster plot. Each bar represents the number of times a target was reached for the corresponding bin. Additionally, the trend line is plotted in red. (Right) Histogram for each subject and target with the number of times a target was reached. Red dots indicate above chance results (confidence 95%). Results show how the time required for reaching the targets decreases as the run goes. Behaviour is similar across subjects, experiments and targets.

During online operation, the device converged to steady performance after 4 targets (Fig. 2c and Fig. S2A), thus rapidly acquiring (quasi) optimal policies in all experiments and reaching desired targets from any starting position. On average users reached 12.38 ± 5.66, 12.46 ± 5.40 and 12.75 ± 6.63 targets per run for Experiments 1, 2 and 3, respectively (see Fig. 4). In contrast, the number of targets reached following a random control policy is 2.27 ± 1.56 for Experiment 1, and 2.32 ± 1.54 for the other experiments. Despite there was a large variability in the convergence rate among subjects, most of them reached a number of targets significantly greater than chance (α = 0.05). (see Fig. 2c, Fig. 4, Supplementary Fig. S2, and Supplementary movie). Figure 2c summarizes the performance in the three experiments when the corresponding device was tested on the same target locations used for training the ErrP classifier. It shows the number of actions required to reach each target within a run. Since the device was initialized at random positions, values were normalized to the initial distance to the target (i.e., a value of one corresponds to optimal performance). For illustration, data from all subjects and all the targets was fitted to an exponential function. For all subjects and targets there was a rapid decrease in the number of actions that converged towards values close to optimal performance; thus reflecting the acquisition of quasi-optimal behaviours (Fig. 4).

In all experiments, the number of learned optimal actions consistently increased as more actions were performed. For experiment 1, the number of optimal actions learned for the visited states was significantly above chance level after 10 actions (false discovery rate (FDR)-corrected one-tailed unpaired t-tests, *p* < 0.05). Furthermore, it consistently increased as more actions were performed (correlation *r* = 0.74, *p* < 1 × 10^−8^). The number of normalized actions required to reach the target converged, to 1.19 ± 0.52 after 9 targets reached, very close to the optimal value (Fig. 2c; red trace). For experiments 2 and 3, the convergence was slower due to the higher number of states and actions: whereas for experiment 1 the number of actions learnt was above chance after 10 actions, for Experiments 2 and 3 it was necessary to execute 15 actions to surpass chance level. (FDR-corrected one-tailed unpaired t-tests, *p* < 0.05). As in Experiment 1, there was also a high correlation between the amount of performed actions and optimal actions learned (*r* = 0.84, *p* < 1 × 10^−8^ for both experiments). The number of normalized actions required to reach the target for experiments 2 and 3 was 2.00 ± 0.76 and 1.97 ± 0.75, slightly worse than for Experiment 1 (Fig. 2c). In summary, all brain-controlled devices were operational almost from the beginning of the run (above chance results after a few actions), improving performance progressively over time (significant correlation between time and number of actions learnt) and approaching optimal behaviour at the end of each run (number of targets reached increasing throughout time, Fig. 4).

### Learning control policies to reach new targets

**Figure 5:**
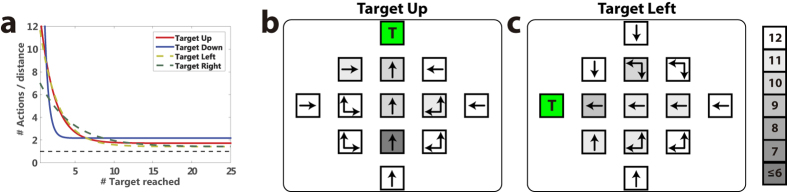
Experiment 3, comparing performance between practice targets (Up and Down), and new targets (Left and Right). (**a**) Normalized number of actions required to reach each target as in Fig. 2. (**b**) Optimal actions per state (denoted by arrows) for a practice target (Up), and (**c**) for a new target (Left). The green square marks the target location. The number of subjects for which the action was correctly learned is color encoded in gray levels.

Experiments with the robot arms demonstrate that control policies can be easily acquired to reach new targets, without the need of retraining the ErrP decoder. Figure 5a shows for the Experiment 3 the number of actions required for practice and new targets. In both cases, the system improves its policy and approaches the optimal behaviour (see Fig. 4 and Supplementary Fig. S2). On average, users reached 14.42 ± 7.81 and 12.54 ± 6.44 targets for experiments 2 and 3 respectively, significantly similar to the ones reached during practice targets (two-tailed paired t-test, p = 0.22 and p = 0.90). Figure 5b,c illustrate the optimal policy for one practice and one new target, respectively. For Experiments 2 and 3, there were no significant differences in the number of optimal actions learned between practice and new targets (FDR-corrected two-tailed paired t-test, *p* = 0.47 and *p* = 0.37, respectively).

Similarly to the practice targets, the number of optimal actions learned was significantly above chance level after 4 and 14 performed actions for Experiment 2 and 3, respectively (FDR-corrected one-tailed unpaired t-tests, *p* < 0.05); and with a high correlation between the amount of performed actions and optimal actions learned (*r* = 0.80, *p* < 1 × 10^−8^ for both experiments). The final number of actions per target for these experiments was 1.81 ± 0.48 and 1.47 ± 1.12. This confirms that the ErrP does not depend on targets, as the ErrP classifier maintains its performance without needing to be retrained for unseen targets.

## Discussion

These experiments illustrate a number of appealing properties associated with the use of error-related brain signals to allow a BMI to teach neuroprostheses suitable motor behaviours. First, we exploit a brain signal naturally elicited by the user, without requiring the explicit learning and execution of *ad-hoc* mental tasks. Moreover, user’s training time is minimal—a calibration session is enough to model the user’s ErrP decoder (25 minutes on average for each subject and experiment). Second, this paradigm makes it possible to achieve tasks in a user-specific manner—the learned control policy depends on the individual user’s assessment. Third, single-trial decoding of ErrP does not need to be perfect to maneuver a neuroprosthesis—it suffices that the ErrP decoder performs statistically above random to learn the motor behaviour. Furthermore, the neuroprosthesis is operational as soon as the accuracy of the ErrP decoder is above chance level—which usually takes minutes as reported here—and keeps adapting indefinitely, as it is the case of human motor control. Finally, and perhaps more importantly, the ErrP is rather independent of the task (e.g., target or action type)—making control of neuroprostheses scalable to more complex tasks since the learning burden is on the robot side.

Scalability is indeed a crucial property of the teaching BMI approach since, as the experimental results demonstrate, ErrPs reflect a common error processing mechanism in the brain across tasks^18^, and this was confirmed by our latest results, which showed that ErrP decoders can generalize across different tasks^26^,^27^. Importantly, error processing information from the brain can be observed using different recording signals such as human electroencephalogram (EEG)^17^,^18^,^19^,^20^,^21^, electrocorticogram (ECoG)^23^ and intracortical recordings^28^. ErrPs have also been reported and decoded in patients with severe motor disabilities^29^. Noteworthy, the development of adaptive mechanisms for BMIs is gaining increased attention^30^,^31^,^32^. ErrPs offer a natural alternative to drive adaptation in the absence of explicit information about goals for both invasive and non-invasive conventional control neuroprosthetic approaches.

As a first demonstration of the proposed paradigm, we have made use of one of the most straightforward and simple RL algorithms, Q-learning. However, this approach—as many other RL algorithms- suffers from two main problems: task generalization and scalability. It is then an open question how the proposed BMI paradigm may generalize across tasks or scale to more complex scenarios. Notwithstanding, current state of the art on reinforcement learning offers very promising alternatives for the tractability of generalization and high-dimensional spaces, such as the use of transfer learning or prior knowledge via demonstration among others^33^.

ErrPs have been exploited to correct and adapt BMIs as well as to improve human-computer interaction^20^,^23^,^24^,^29^. Along this line, a possibility that has been explored in rodents is to extract information from the reward-processing brain areas (i.e., nucleus accumbens)^22^ for RL-based adaptation of the BMI decoder^34^. Here we go beyond this BMI adaptation framework and, extending our previous 1D works^21^,^35^, demonstrate for the first time in humans how the teaching BMI paradigm enables the acquisition of suitable control policies, scales to neuroprostheses with a task complexity similar to state-of-the-art BMIs, and generalizes across different targets.

In the experiments reported here, it is assumed that the neuroprosthesis owner wishes to initiate a voluntary, goal-directed movement whose low-level execution is delegated to subcortical, spinal cord and musculoskeletal structures. In our case, this lower level of motor control is emulated by an intelligent controller able to learn and to reuse control policies via ErrPs. Although a full demonstration of this extension to the teaching BMI approach remains to be proven, evidence suggests its feasibility. Firstly, cortical cognitive signals indicating goals^3^,^12^, self-paced onset of movements^13^, or anticipation of purposeful actions^36^,^37^ can be decoded at the single-trial level. Secondly, several control policies can be learned as demonstrated here and stored to form a repertoire of motor behaviours (i.e., to reach different targets within the environment). Note that, while in the current manuscript one control policy was associated with a specific target, one target could have several ways of being reached depending on the user’s preferences. Once the control policies are stored, the decoding of the error-related signals can be exploited to infer the desired behaviour from this repertoire rather than being used to learn a new control policy. This possibility has been recently explored in^38^.

We postulate that the combination of all these sorts of cognitive brain signals would be sufficient for chronic operation of neuroprostheses, whose range of tasks may change over time. Such a possibility is critical for patients—especially if suffering from neurodegenerative diseases—as they must rely upon neuroprostheses for extended periods of time. Despite remaining hurdles such as large clinical studies, further research will uncover additional cognitive brain signals that will enrich this initial basic set, thus enlarging the repertoire of decision-making processes available for natural, intuitive control of neuroprostheses to perform goal-directed movements and bringing BMI closer to therapeutic reality.

## Methods

All experiments were carried out in accordance with the approved guidelines. Experimental protocols were approved by the Commission Cantonale (VD) d'éthique de la recherché sur l'être humain (protocole 137/10). Informed written consent was obtained from all participants that volunteered to perform the experiments.

### Subjects and data recording

Twelve able-bodied volunteers (four females, 23–24 years) participated in the study. EEG signals were recorded from 16 active electrodes located at Fz, FC3, FC1, FCz, FC2, FC4, C3, C1, Cz, C2, C4, CP3, CP1, CPz, CP2, and CP4 (10/10 international system). The ground was placed on the forehead (AFz) and the reference on the left earlobe. EEG was digitized at 256 Hz, power-line notch filtered at 50 Hz, and band-pass filtered at [1, 10] Hz. To reduce signal contamination, participants were also asked to restrict eye movements and blinks to indicated resting periods.

### Experimental setup

All participants performed three experiments of different complexity, evaluated using the NASA-TLX questionnaire (28.44 ± 14.01, 42.33 ± 23.31 and 46.92 ± 22.19 for Experiments 1 to 3) in a different age-matched set of nine subjects. Each experiment was carried out on a different day, lasting around 2.5 hours. The time elapsed between two consecutive experiments was 17.58 ± 10.09 days. In all experiments, subjects were instructed to monitor the device while it tried to reach a target (only known by the subject) and to assess whether the device actions were correct or incorrect. Each experiment was divided into two phases: training and online operation of the neuroprosthesis. Each phase was composed of several runs, each run consisting of 100 device actions. During each run the target location remained fixed and, whenever the device reached that location, its position was randomly reset to a location at least two positions away from the target, see Fig. 2a.

The training phase aimed at building a classifier able to detect error potentials. In the initial runs, the device performed erroneous actions with a fixed probability (20%). For all experiments, two target locations were used in this phase. After each run, all the collected data was used to train the ErrP classifier^26^,^27^. Once the decoding accuracy was above 80%, or four runs were elapsed, an additional run was executed where the output of the classifier was used to adapt the device controller using RL (see below). Thus, in this RL run the error probability was variable. If the accuracy in this RL run was below random, the classifier was retrained with additional RL runs until the criterion (accuracy above random) was reached. Subjects needed a median (±mean absolute deviation) of 1.00 ± 0.51 additional RL training runs. The mean duration of the entire training phase for all subjects and experiments was 25 minutes. The maximum length was 45 minutes.

In the online operation phase, the information decoded from the EEG (indicating whether the subject considered the action as correct or erroneous) was used as a reward signal to learn the behaviour through RL. One run was performed per target location (2 runs in the case of Experiment 1, and 4 runs for Experiments 2 and 3). In the last two experiments we tested the generalization capabilities of the proposed approach by including target locations that were not used in the training phase. In all RL runs, the device controller was initialized to a random behaviour where all actions at a given location are equiprobable.

#### Experiment 1: Moving Cursor^21^ (Fig. 2a, Left)

Participants faced a computer screen showing a horizontal grid with nine different positions (states; c.f., squares in Fig. 2a), including one blue moving cursor (device) and one red square (target). The cursor could execute two actions: move one position to the left or to the right. When the cursor was at the boundaries (i.e., at the left-or right-most states), actions that moved it out of the state space were not allowed. The time between two consecutive actions was drawn randomly from a uniform distribution within the range [1.7, 3.0] s. Only states at the left-most and right-most positions were used as targets.

#### Experiment 2: Simulated Robotic Arm (Fig. 2a, Center)

Subjects faced a computer screen displaying a virtual robot (device). We simulated a Barrett whole arm manipulator (WAM) with 7 degrees of freedom using the RobotToolkit framework developed by the LASA laboratory at EPFL (http://lasa.epfl.ch). The robot could place its end-effector at 13 different positions (states; c.f., orange squares in Fig. 2a), with one position in green (target). It could perform four actions: moving one position to the left, right, up, or down. As before, when the device was at a boundary state, actions that moved the robot out of the state space were not allowed. In contrast to the first experiment, the robot movements between two states were continuous, lasting ~500 ms. The time between two consecutive actions was randomly distributed within the range [2.5, 4.0] s. During the training phase, the targets were located at up-and down-most positions (i.e., practice targets). For the online operation phase, the up-, down-, left-, and right-most positions were tested as targets.

#### Experiment 3: Real Robotic Arm (Fig. 2a, Right)

This experiment followed the same design as Experiment 2 but involving a real robotic arm (Barrett WAM). The robot was two meters away from the user and was pointing at states on a Plexiglas transparent panel between the two. The distance between two neighbor states was 15 cm.

### ErrP classifier

EEG signals were spatially filtered using common average reference and downsampled to 64 Hz. Features were extracted as the signal from eight fronto-central channels (Fz, FCz, Cz, CPz, FC1, FC2, C1, and C2) within a time window of [200, 800] ms from the device movement onset and concatenated to form a vector of 312 features. These vectors were normalized and decorrelated using principal component analysis^39^. The most discriminant features were selected based on the *r*^*2*^ score using a five-ten-fold cross-validation on the data of the training phase. On average, 36 ± 13 features were selected per subject. ErrPs were decoded using a linear discriminant analysis (LDA) classifier.

To assess the statistical significance of the ErrP classifier accuracies during online operation, we compute the chance levels (α = 0.05) according to the available number of trials using the binomial cumulative distribution^40^. The estimated chance levels were 56% for Experiment 1 and 54% for Experiments 2 and 3.

### Reinforcement learning (RL) with ErrPs

The RL strategy^25^ was modeled by a Markov decision process, denoted by the tuple {*S, A, r, γ*} with *S* being the state space (the possible positions of the device), and *A* the action space (the possible actions of the device). The reward value *r* represented the goodness of the executed action at a given state and *γ* is a time discount factor. The goal of RL was to obtain a control policy *π:S* *→* *A* mapping the state space into the action space (i.e., which action had to be performed at each state) so as to maximize the expected return *R* = Σ^∞^_k=0_ γ^*k*^*r*_*k+1*_ at time *k*. The RL implementation was the Q-learning iterative algorithm^25^:


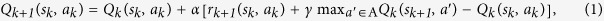


where *k* is the current step and α is the learning rate. Parameters γ and α were set empirically to 0.4 and 0.1, respectively. During online operation, at time *k*, the device executes an action *a*_*k*_ that takes it from state *s*_*k*_ to state *s*_*k+1*_, according to its current policy. The output of the ErrP classifier is then used to obtain the reward value *r*_*k+1*_*(s*_*k*_*, a*_*k*_); it takes a value of −1 if the action is classified as error, otherwise is set to +1. This reward was used to update the RL policy after each action. All Q-values were set to zero at the beginning of each run (*k* = 0), corresponding to a random control policy. At the end of the run, the final policy π was computed as the policy that, at each state *s*, always followed the action *a′* with the maximum Q-value, π(*s*) = arg max_*a*′∈A_ Q(*s, a′*).

At each step *k*, an ε-greedy strategy was used to select the next action *a*_*k*_ to be executed. This policy selected the action with highest Q-value (best action) for (100 − ε) % of the times, while a random action was selected the remaining times. The experiments started with a completely exploratory behaviour (ε = 100%), and every time an exploratory action was chosen ε was decreased by a constant factor (5%) until reaching a minimum value (20%) to always maintain a small percentage of exploration.

### Acquisition of control policies (or behaviours)

We evaluated the acquisition of control policies using the number of optimal actions (i.e., those leading to the target location, c.f. arrows in Fig. 5b, and Fig. 5c) learned by the controller at a given time. Only states already visited were considered in this measure. We also compared the number of optimal actions learned to the chance level, computed as the number of actions learned with random rewards (i.e., ±1 with equal probabilities). Statistical tests were corrected with the false discovery rate, FDR^41^.

Learning of the control policies was also assessed in terms of the number of actions required to reach the target location within a run. To account for the different initial states, the number of actions is divided by the initial distance to the target. For illustration purposes, we fitted the data of each experiment to an exponential curve, *y* = *a* + *be*^*−cx*^, where y is the normalized number of actions required to reach the target for the *x*-th time (c.f., Figs 2c and 5a and Supplementary Fig. S2).

### Analysis of ocular artifacts

We assessed the possibility of EEG signal contamination by movement-related ocular artifacts. We computed the grand average ERPs (correct and error) of all channels separately for each different action (moving left, right, up, or down). No substantial differences were found among these ERPs, suggesting little influence of eye movements. This is illustrated in Supplementary Fig. S1 that shows the averages of three fronto-central electrodes (FC3, FCz and FC4), separated by assessment (correct or error) and movement direction (left, right, up or down). As can be seen, the differences among assessments were larger than the differences among directions. This is consistent with previous studies that found no influence of this type for Experiment 1^20^,^21^.

To evaluate the existence of statistical differences due to both assessments and movement directions, we performed 2 (factor assessment: error or correct) × 4 (factor movement direction: left, right, up or down) within-subjects ANOVAs on the values of the most prominent positive and negative peak amplitudes of the grand averages of channel FCz (note that for Experiment 1 the ANOVA was 2 × 2 since there were only two possible movement directions). When needed, the Geisser-Greenhouse correction was applied to ensure sphericity. The assessment and direction main effects and the assessment × direction interaction were studied.

Regarding the main effects, statistical differences were found for the assessment for all the experiments, for the positive (F_1,11_ = 17.277, *p* = 0.002, F_1,11_ = 15.567, *p* = 0.002, and F_1,11_ = 14.202, *p* = 0.003 for Experiments 1 to 3) and negative (F_1,11_ = 10.087, *p* = 0.009, F_1,11_ = 14.658, *p* = 0.003, and F_1,11_ = 11.581, *p* = 0.006) peaks. On the contrary, no significant differences were found for the direction main effect (*p* > 0.1). Regarding the assessment × direction interaction, significant differences were found during Experiment 2 (F_3,33_ = 3.721, *p* = 0.02 and F_3,33_ = 3.903, *p* = 0.02 for the positive and negative peak); and during Experiment 3 for the negative peak (F_3,33_ = 3.461, *p* = 0.03) but not for the rest of the cases (*p* > 0.35). These results indicated that the largest differences on the potentials were due to the different assessments (error/correct), whereas the movement directions of the device affected less the potentials.

## Additional Information

**How to cite this article**: Iturrate, I. *et al*. Teaching brain-machine interfaces as an alternative paradigm to neuroprosthetics control. *Sci. Rep*. **5**, 13893; doi: 10.1038/srep13893 (2015).

## Supplementary Material

Supplementary Information

Supplementary Movie S1

## Figures and Tables

**Figure 1:**
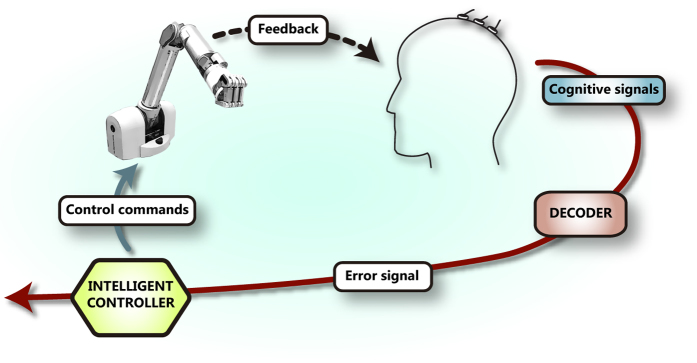
Teaching BMI paradigm. In contrast with the standard control approach, in this paradigm users assess the actions performed by the neuroprosthesis as erroneous or correct. This information is decoded from the user’s brain signals, and exploited by the reinforcement learning algorithm embedded in the neuroprosthesis controller to learn appropriate motor behaviours (or control policies) to perform different reaching tasks. See also Supplementary movie.
