# Supplementary material for: Teaching brain-machine interfaces as an alternative paradigm to neuroprosthetics control
Source: Sci Rep. 2015 Sep 10;5:13893. doi: 10.1038/srep13893 (PMC4564803; doi:10.1038/srep13893)
Supplement: Supplementary Information [file srep13893-s1.pdf]

SUPPLEMENTARY INFORMATION

Teaching brain-machine interfaces as an alternative paradigm to neuroprosthetics control

Authors: Iñaki Iturrate[1,2], Ricardo Chavarriaga[2], Luis Montesano[1], Javier Minguez[1], and José del R. Millán[2*]

Affiliations:

[1]Instituto de Investigación en Ingeniería de Aragón, Dpto. de Informática e Ingeniería de Sistemas, Universidad de Zaragoza, Spain

[2]Chair in Non-Invasive Brain-Machine Interface, Center for Neuroprosthetics & Institute of Bioengineering, Ecole Polytechnique Fédérale de Lausanne, CH-1015 Lausanne, Switzerland

Supplementary Information

Fig. S1. Grand averaged ERPs.

Fig. S2: Acquisition of control policies for all experiments and targets.

Table S1. Electrode locations used as factors for the ERP statistical analysis.

Movie S1. Demonstration of the teaching BMI paradigm.

Methods

Analysis of error-related potentials waveforms

We analyzed variations in the grand average EEG potentials for both conditions, i.e., erroneous and correct device actions (see Fig. 2B and Supplementary Fig. S1, left panel). To this end we performed a statistical analysis on the difference ERP (error minus correct condition) for all electrodes in the time window [-200, 1000] ms, t =0 ms being the instant when the device starts to move. Only signals from the training runs —having a constant error-rate (20%)— are used in this analysis. These runs yielded between 200 and 400 trials for each subject.

A 3 (brain area: frontal, central or centro-parietal electrode locations) x 3 (left, midline or right locations) x 3 (experiments) within-subjects ANOVA was performed on the peak amplitudes and latencies of the difference average. Each group is summarized in Supplementary Table 1. When needed, the Geisser-Greenhouse correction was applied to assure sphericity. Pairwise post-hoc tests with the Bonferroni correction were computed to determine the differences between pairs of experiments.

We mainly found significant effects on the latency but not the amplitude of the difference potential. Latency increased with the complexity of the experiments. The type of experiment significantly affected the latencies of both the positive ($F_{2,22}$ = 41.594, p =3×10^{-8}) and negative peaks of the difference ERP ($F_{2,22}$ =7.522, p =0.003). The brain area also affected the latencies of the positive peak ($F_{1.32,14.55}$ = 14.175, p =0.001) but not the negative one $F_{2,22}$ =0.911, p =0.417). Similarly, the hemisphere affected the latency of the positive peak ($F_{2,22}$ =5.279, p = 0.013), but not the latency of the negative one ($F_{2,22}$ =1.711, p =0.204). No significant interactions were found.

For the positive peak latencies, post-hoc pairwise tests revealed significant differences between experiments 1 and 2 (p =0.0001), and between experiments 1 and 3 (p =0.0001), and close to significant difference between experiments 2 and 3 (p =0.068). For the negative peak latencies, there were significant differences between experiments 1 and 3 (p =0.009), but not between experiments 1 and 2 (p =0.357) or experiments 2 and 3 (p =0.122).

In contrast, the amplitudes of the positive and negative peaks were not significantly affected by the experiment ($F_{2,22}$ =0.124, p =0.884 and $F_{2,22}$ =2.304, p =0.123, respectively) nor the brain area ($F_{1.19,13.08}$ = 1.227, p =0.737 and $F_{1.32,14.47}$ =0.071, p =0.857). The laterality significantly affected the positive peak amplitude ($F_{2,22}$ =4.556, p =0.022), and was close to significance for the negative peak ($F_{2,22}$ =3.425, p =0.051). As in the case of the peak latencies, no significant interactions were found.

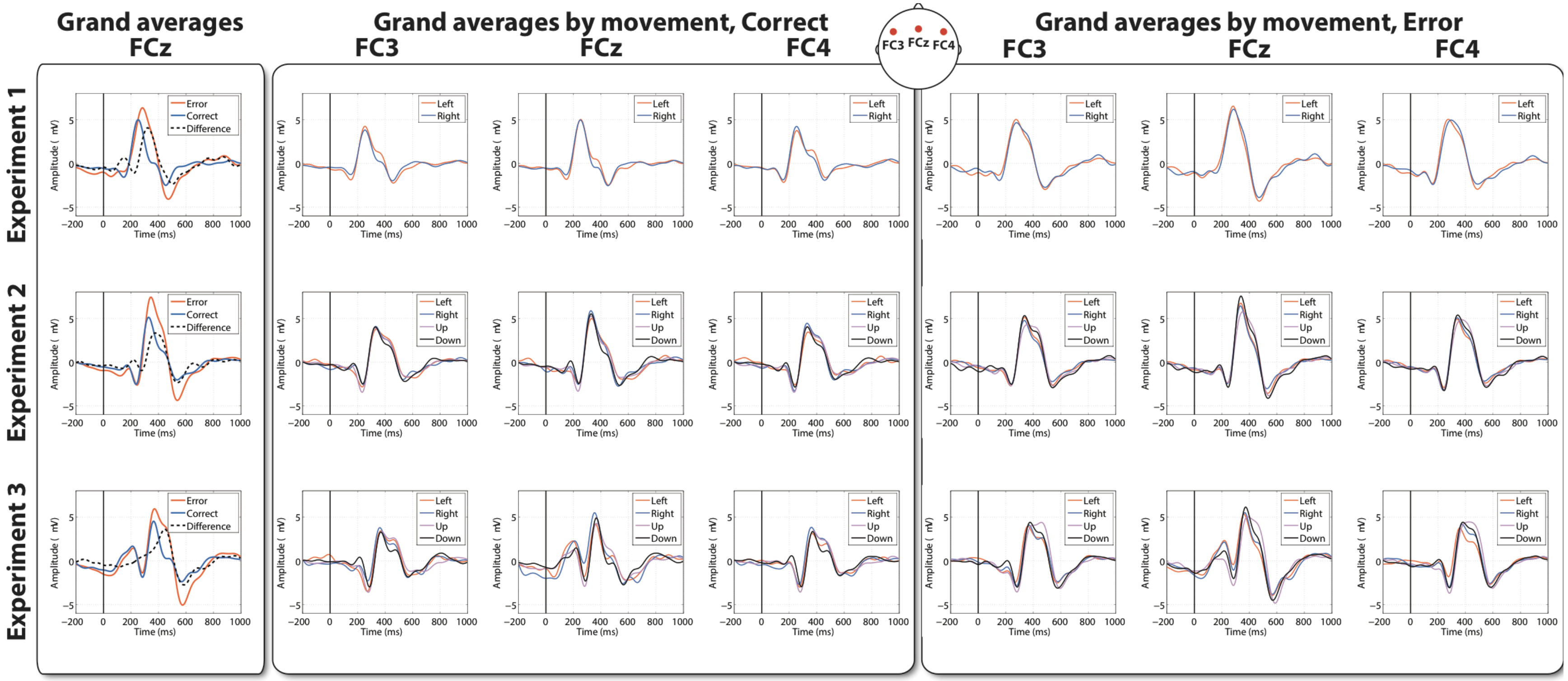

Fig. S1. Grand averaged ERPs. Grand averaged ERPs in channels FC3, FCz, and FC4 over all subjects (N=12). Each row corresponds to a different experiment. Left panel displays the grand averages at FCz of correct (blue), error (red), and difference (black). Center and right panels illustrate the grand averages for correct and erroneous assessments of each movement direction (left, right, up or down), respectively. These two panels show that the averaged signals are very similar across all directions, reducing the possibility that the direction of robot movements may have a systematic influence on the ErrP classification process.

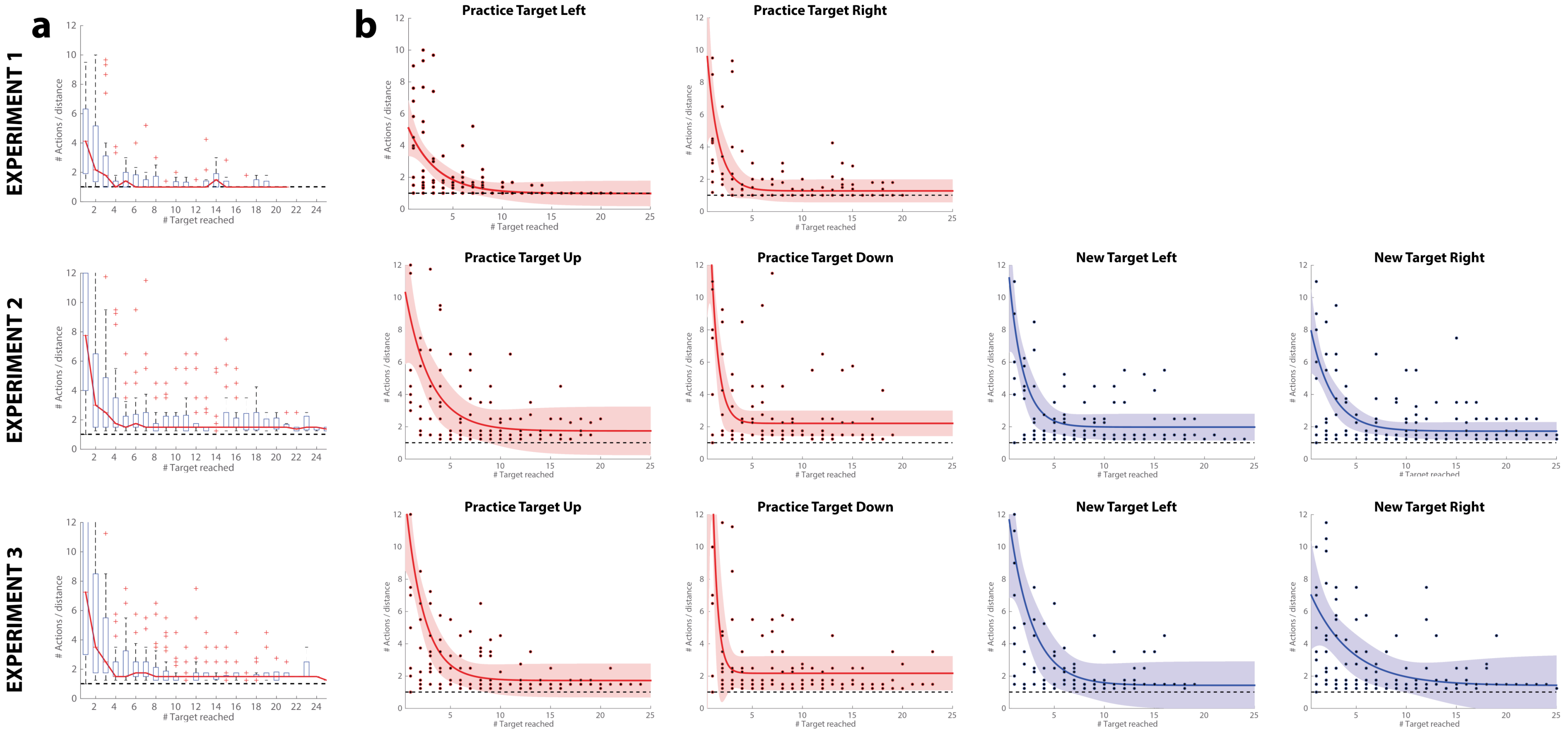

Fig. S2. Acquisition of control policies. (a) Acquisition of control policies for all the targets, and each experiment separately, as a bar plot each time a target was reached. Additionally, the median line is also shown in red. (b) Acquisition of control for each experiment and target. Dots represent the normalized number of actions required to reach a target location during an online run (all subjects together). The continuous line corresponds to an exponential fitting ($y = a + be^{-cx}$) of the data. The shadowed area corresponds to the 95% confidence interval of the fitting. A consistent decrease in the number of actions required to reach the goal is observed for all targets. Moreover, no difference is observed in experiments 2 and 3 between practice (up and down, red) and new targets (left and right, blue).

Table S1. **Electrode locations used as factors for the ERP statistical analysis**. Each row corresponds to the brain areas, while columns correspond to the laterality.

	**Left**	**Midline**	**Right**
**Frontal**	FC3, FC1	Fz,FCz	FC2,FC4
**Central**	C3,C1	Cz	C2,C4
**Centro-parietal**	CP3,CP1	CPz	CP2,CP4

Movie S1. **Demonstration of the teaching BMI paradigm.** The movie demonstrates the online operation of our teaching BMI paradigm with a real arm robot executing a reaching task. The user monitors the performance of the robot arm that has no knowledge about the target location (green square). Error-related potentials (ErrPs) are elicited whenever an executed action does not match the user's expectations. These ErrPs are decoded online as shown in the bottom-left part. The outcome of this decoding is used as a reward signal for a reinforcement learning algorithm that updates the control policy. This policy is shown in the upper-right panel, where arrows show the estimated optimal action for each state. As observed, acquisition of (quasi-)optimal actions is very fast. Trajectories are initially random and progressively become straight throughout the run (100 actions).
